# The Reality of Active Targeting in Nanomedicine: Promise Versus Performance

**DOI:** 10.1002/cbic.70475

**Published:** 2026-07-24

**Authors:** Francesco Cellesi

**Affiliations:** ^1^ Department of Chemistry Materials and Chemical Engineering “Giulio Natta” Politecnico di Milano Milan Italy

**Keywords:** active targeting, biological barriers, multivalent interactions, nanomedicine, spatiotemporal control

## Abstract

Active targeting in nanomedicine aims to enhance therapeutic efficacy by functionalising nanocarriers with ligands that selectively bind biological targets. Despite extensive research, clinical translation has remained limited, with most approved systems relying on antibodies or passive delivery mechanisms rather than complex targeted nanoparticles. This perspective critically examines the gap between the conceptual promise of active targeting and its practical performance. Drawing comparisons with biological systems, we highlight that natural targeting relies on dynamic, multi‐step and energy‐dependent processes rather than simple ligand–receptor interactions. Current nanomedicine approaches are constrained by an oversimplified view of targeting, neglecting key factors such as multivalency, spatial organisation, kinetics and biological barriers. Future progress requires a shift towards systems‐level design, integrating spatiotemporal control, adaptive materials and computational modelling. Ultimately, we propose a design‐by‐architecture paradigm, in which targeting is encoded into nanomaterial structure, enabling programmable and biologically guided delivery.

## Graphical abstract

Schematic representation of a multi‐functional nanocarrier decorated with diverse ligands. Ligand type, density and spatial organisation collectively determine targeting behaviour, highlighting the complexity of active targeting beyond simple ligand–receptor interactions.

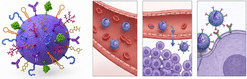

## 1 Introduction

**FIGURE 1:**
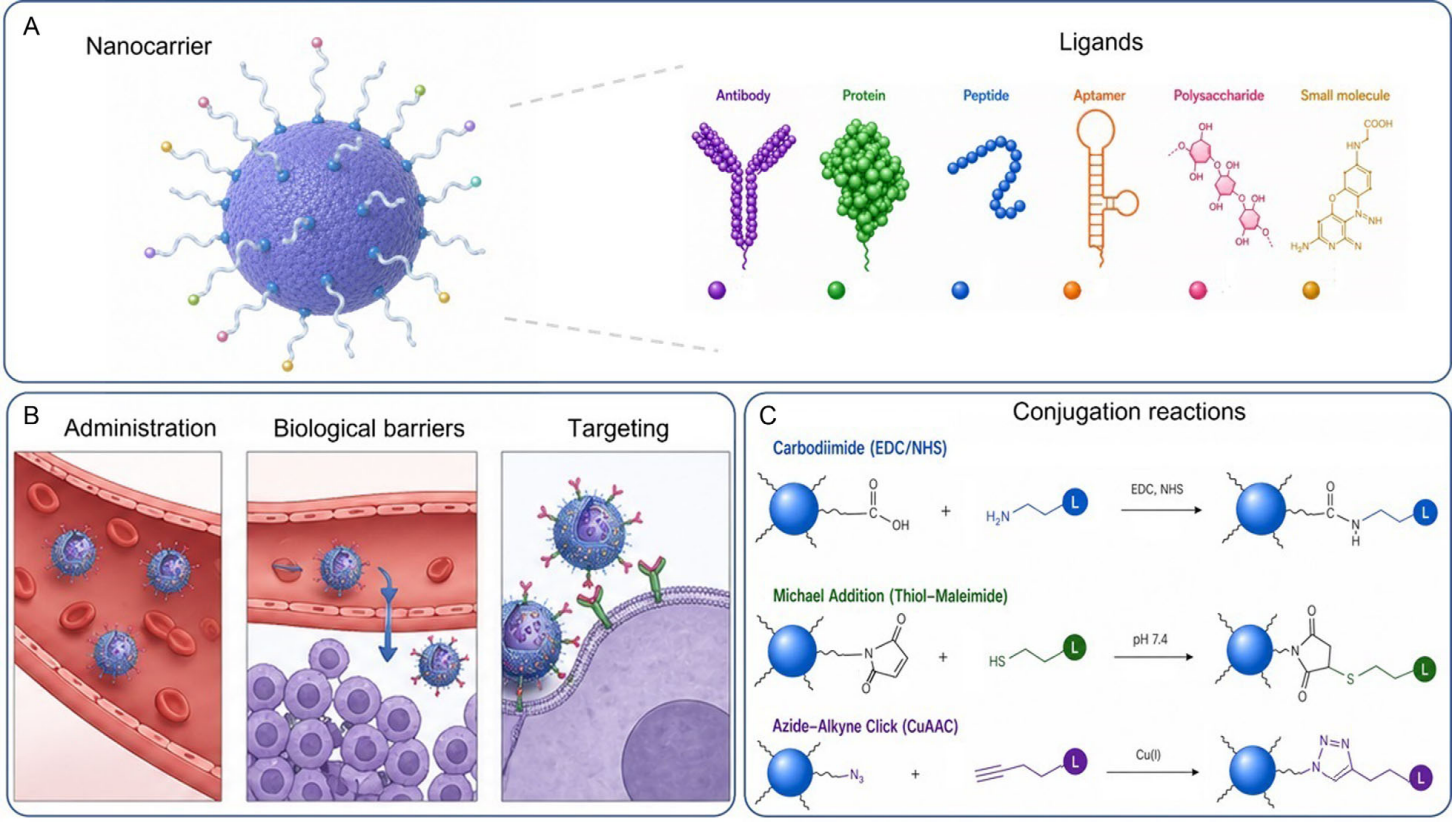
(A) Nanocarriers designed for active targeting are functionalised with specific ligands, such as antibodies, proteins, peptides, aptamers, polysaccharides or small molecules. (B) After administration, these nanocarriers navigate multiple biological barriers to reach target cells or tissues and interact with specific receptors or other molecular components. (C) Common conjugation strategies used to attach the selected ligand to the nanomaterial.

Targeting remains a major challenge in biomedical technologies, particularly in drug delivery and in the development of nanomaterials for therapeutic applications [1, 2]. In nanomedicine, active targeting refers to the functionalization of nanoscale drug carriers with ligands that enable selective binding to specific cells or tissues, typically via receptor–ligand interactions. This strategy aims to enhance therapeutic efficacy while reducing side effects by minimising off‐targeting commonly associated with passive distribution and limited selectivity. Current strategies predominantly rely on the conjugation of ligands (typically antibodies, peptides, nucleic acids, saccharides) either to the active molecule or to the surface of nanocarriers [3] (Figure 1). Although ligand–receptor interactions are fundamental mechanisms widely exploited in nature for cellular communication, signalling and regulation across diverse biological contexts [4], active targeting in nanomedicine remains limited by poor selectivity, limited ability to overcome biological barriers, susceptibility to immune recognition and insufficient control over nanomaterial properties, ultimately resulting in inadequate targeting performance [5, 6].

General considerations highlight key limitations in this research field. Despite substantial investments over the past two decades, targeted nanodelivery has largely fallen short of expectations. An examination of nanomedicine products that have received marketing authorisation shows that actively targeted systems are predominantly based on therapeutic antibodies or simple conjugation of antibodies to low‐molecular‐weight therapeutics or imaging agents [7, 8]. Other approved nanomedicines include PEGylated therapeutic proteins that lack active targeting capabilities. More complex nanoparticulate systems currently on the market are mainly liposomal formulations, which are not functionalised with targeting ligands and whose efficacy largely relies on passive accumulation mechanisms or the solubilisation of highly hydrophobic drugs [8]. Other nanotherapeutic approaches have struggled to achieve efficient targeting and have consistently failed to meet the stringent requirements of clinical translation. This has had significant consequences. In 2019, the U. S. National Cancer Institute (NCI) announced that it would not renew funding for its Centers of Cancer Nanotechnology Excellence (CCNEs), following more than 15 years of investment exceeding $300 million [9]. This decision reinforced the perception that nanomedicine may have been driven more by hype than by tangible clinical impact, ultimately undermining confidence in its potential as a transformative therapeutic platform. Despite this, nanomedicine has delivered notable successes, particularly in vaccine technology. RNA‐based COVID‐19 vaccines, which rely on lipid nanocarriers, represent a clear example [10]. The contrast between these outcomes lies in the underlying targeting mechanisms: RNA‐based nanovaccines exploit innate immune recognition and cellular uptake of foreign material, thereby bypassing the need for precise ligand–receptor‐mediated targeting.

### 1.1 Examples of Active Targeting in Approved Medicinal Products

Some key considerations emerge. One might argue that active targeting is not the appropriate strategy for the development of future drug delivery systems and that it offers limited potential to enhance therapeutic efficacy. However, biological systems provide abundant evidence to the contrary, as the body extensively relies on active targeting mechanisms to mediate transport and communication across different spatial scales. At the molecular level, hormones, cytokines and other soluble mediators travel through body fluids to act on distant target cells, while antibodies circulate and selectively recognise specific antigens. Extracellular vesicles (EVs) transport proteins, lipids and nucleic acids between cells, enabling targeted intercellular communication. At the cellular level, immune cells migrate through blood and tissues towards sites of infection via chemotaxis and homing signals, while stem and progenitor cells are recruited to areas of injury to support tissue repair, ensuring coordinated and spatially controlled targeting. Efforts to mimic these natural targeting mechanisms have led to the development of successful therapeutic technologies, although not necessarily within nanomedicine. In particular, antibody‐based therapies have demonstrated substantial clinical impact and chimeric antigen receptor (CAR) T‐cell therapy represents a clear example of effective active targeting in the context of cell‐based therapies. In this approach, a patient's T cells are genetically engineered to express CARs that recognise tumour‐specific antigens and trigger cytotoxic responses [11, 12]. CAR T therapy has been highly successful in hematological malignancies, where tumour cells are accessible and display relatively uniform antigen expression [13]. In contrast, its efficacy in solid tumours remains limited due to multiple barriers, including poor T‐cell infiltration, antigen heterogeneity and an immunosuppressive tumour microenvironment that inhibits T‐cell activity and reduces effectiveness [14]. These limitations highlight that overcoming biological barriers remains a major challenge for active targeting‐based technologies. Building on these general considerations, active targeting is hereafter examined from the perspective of nanomaterial design and engineering, and the biological mechanisms that ultimately govern targeting performance.

### 1.2 Current Chemistry

Ligand conjugation to nanomaterials relies on a relatively limited set of robust and well‐established chemistries that enable the covalent attachment of biomolecules to nanocarriers [15, 16] (Figure 1). The most widely used approaches are favoured for their high efficiency, reproducibility and compatibility with biological conditions. Classical strategies involve amine and thiol chemistries: lysine residues react with NHS‐esters to form amide bonds, while cysteine thiols undergo Michael‐type addition with electron‐poor olefins (e.g., maleimides, acrylates, vinyl sulphones) to yield stable thioether linkages. PEG‐based linkers are also frequently employed to enhance solubility, reduce steric hindrance and prolong circulation time. Click chemistry reactions are also widely used, including copper‐catalysed azide–alkyne cycloaddition (CuAAC), as well as copper‐free alternatives such as strain‐promoted azide–alkyne cycloaddition (SPAAC) and inverse electron‐demand Diels–Alder (IEDDA), which enable rapid and selective conjugation under mild, physiologically compatible conditions. Enzymatic and site‐specific strategies, including the incorporation of non‐natural amino acids or glycan modification, further improve conjugate homogeneity and targeting precision. Overall, these methods provide reliable routes to stable, ligand‐functionalised nanocarriers, often achieving near‐quantitative conversion and efficient product isolation. However, analytical challenges remain, particularly in fully characterising materials with low degrees of functionalisation [17]. Together with cost and process complexity, some of these techniques may pose challenges for scalability, regulatory approval and good manufacturing practice compliance. Nevertheless, conjugation chemistry can be considered a mature field and is unlikely to represent the primary limitation in the clinical translation of targeted nanomedicines.

### 1.3 Type of Ligands

An effective targeting strategy requires the right choice of the ligand to achieve selective binding, for example, towards receptors overexpressed on diseased cells, enhancing cellular uptake and drug accumulation [16, 18]. Major ligand classes include antibodies, for example, the ones which provide high specificity towards tumour‐associated antigens (e.g., HER2, EGFR), enabling strong receptor‐mediated targeting. Peptides and proteins, such as RGD or transferrin, bind integrins or transferrin receptors and improve tumour penetration and internalisation. Polysaccharides like hyaluronic acid target CD44 receptors, commonly overexpressed in cancer cells, while saccharide ligands (e.g., galactose, N‐acetylgalactosamine) enable liver‐specific targeting via asialoglycoprotein receptors. Small molecules such as folic acid and biotin bind nutrient receptors upregulated in tumours, offering simple and effective targeting strategies. Additionally, aptamers, short nucleic acid sequences, provide high affinity and specificity similar to antibodies but with improved stability and lower immunogenicity [3]. Overall, these ligands enhance selective recognition, promote receptor‐mediated endocytosis and improve therapeutic potential in targeted drug delivery systems. However, the targeted receptors are rarely exclusive to a single cell type, and their overexpression does not ensure complete selectivity, often leading to off‐target interactions. Furthermore, the presence of targeting ligands can increase immune recognition, promoting opsonisation and clearance, thereby limiting effective delivery [19].

## 2 Active Targeting in Our Body

From a biology point of view, ligand–receptor binding is a fundamental mechanism extensively exploited by nature for cellular communication, signalling and regulation across diverse biological contexts. Here, we report some key examples, with their mechanism and limits of applicability.

### 2.1 Antibody Transport

Antibodies are a classic example of active targeting because they bind specific antigens with high affinity, but this selectivity occurs after passive distribution, making their targeting molecular rather than tissue‐directed. Antibody targeting is most efficient in well‐accessible, highly perfused environments such as blood, hematological tissues and inflamed sites, where targets are exposed and barriers are minimal, while it is less effective in protected or dense tissues like the brain or solid tumours [20, 21]. Antibodies cross biological barriers mainly through receptor‐mediated transcytosis, especially via the neonatal Fc receptor (FcRn), which transports IgG across endothelial and epithelial cells while extending its half‐life [22]. These mechanisms enable controlled entry into tissues but operate with limited efficiency and capacity. Although antibodies exhibit high molecular specificity, their tissue distribution depends on receptor expression, barrier properties and transport dynamics, resulting in selective yet non‐exclusive targeting rather than precise delivery.

### 2.2 EVs Targeting

Experimental evidence shows that EVs can mediate long‐distance systemic signalling and exhibit non‐random biodistribution [23]. In vivo studies, particularly in cancer, indicate organ‐biased accumulation linked to surface molecules such as integrins, with functional effects in recipient tissues [24]. However, high organ or cell‐type selectivity is generally not achieved. Systemically circulating EVs are rapidly cleared, predominantly accumulating in the mononuclear phagocyte system (mainly in liver, spleen and lungs) [25]. Thus, EV targeting is best described as biased enrichment rather than precise homing. This limited specificity, combined with EV heterogeneity and technical constraints in tracking, highlights major challenges and motivates ongoing efforts to enhance targeting efficiency.

### 2.3 Virus Targeting

Although viruses represent a risk for human health, they are very interesting to study from the point of view of the mechanism they use to infect our cells. Viruses are generally more selective than EVs but remain far from perfectly precise. Their targeting arises from molecular compatibility and tissue accessibility rather than active navigation, with receptor–ligand interactions serving as the primary determinant of cell entry [26]. While this restricts infection to certain cell types, receptor expression across multiple tissues limits exclusivity. Consequently, viral tropism reflects relatively high yet non‐absolute selectivity, shaped by receptor distribution, exposure route and off‐target infection, best described as constrained permissiveness rather than precise targeting.

### 2.4 How Active Targeting in Nature Overcome Biological Barriers

In biological systems, crossing barriers is not achieved by targeting alone but through active, regulated transport mechanisms. Nature combines molecular recognition with energy‐dependent processes such as receptor‐mediated transcytosis, carrier transporters and cell‐mediated delivery. Cells can also actively migrate across barriers using chemotaxis, remodel their environment by degrading extracellular matrix, and temporarily modulate tight junctions to allow passage. These mechanisms are highly coordinated and context‐dependent, since entry is permitted only under specific conditions. Importantly, many of these processes involve living cells acting as carriers, not just the passive movement of molecules. The key insight is that biological barrier crossing relies on the integration of recognition, transport and environmental control, rather than a single targeting interaction. In other words, binding to a target is only one step; successful delivery requires active navigation and regulated passage through multiple barriers.

## 3 Need of Spatiotemporal Biological Signals

**FIGURE 2:**
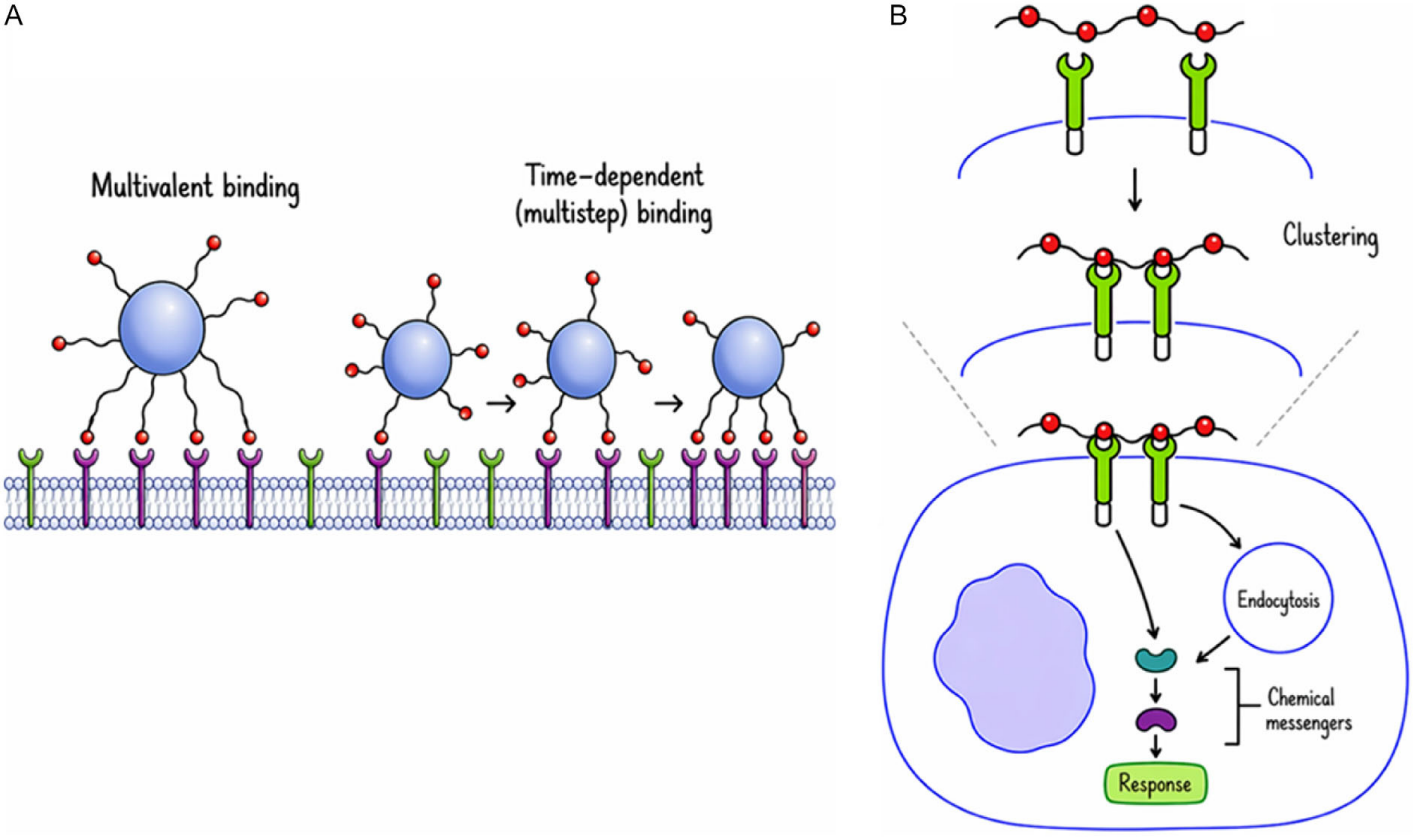
(A) Multivalent binding of ligand‐decorated nanocarriers enables simultaneous interactions with multiple receptors, leading to time‐dependent, multi‐step binding processes that increase overall avidity. (B) Ligand–receptor interactions can induce receptor clustering at the cell membrane, triggering intracellular signalling and endocytosis, ultimately leading to a biological response.

Building on these considerations, effective active targeting requires precise spatiotemporal control of biological signals. While targeting enables localisation to specific sites, therapeutic outcomes depend on the timing, sequence, and concentration of multiple signals, reflecting the dynamic and multi‐step nature of biological systems. Limitations of active targeting in nanomedicine often come from a simplified ‘lock‐and‐key' view of ligand–receptor interactions, which does not capture the full complexity of biological systems. Multivalent interactions play a central role by enhancing binding strength (avidity) and promoting stable but reversible binding, thus improving retention at target sites (Figure 2A). However, their effectiveness depends strongly on ligand density [27], which exhibits a non‐linear relationship with targeting efficiency: insufficient density leads to weak interactions, whereas excessive density can induce steric hindrance, reduced ligand flexibility and increased immune recognition. Targeting depends on both ligand number per particle and receptor density, and only a fraction of ligands are functionally accessible [28]. In ligand–receptor mediated systems, binding can be tuned to occur selectively within a defined window of receptor densities, with minimal interaction outside this range [29]. Accordingly, increasing ligand density does not necessarily improve targeting; instead, optimal and often low densities, combined with controlled spatial organisation, enhance selectivity by balancing accessibility, multivalent interactions and tissue penetration [30]. Moreover, nanoparticle‐induced receptor clustering (Figure 2B) can significantly influence cellular uptake and signalling, representing an important but often underexploited mechanism [31]. Targeting is therefore governed not only at the molecular level but also at a mesoscale level, where ligand organisation, clustering and spatial arrangement (topology) regulate receptor engagement [32]. Rather than maximising ligand affinity, an optimal balance between affinity and multivalency is required to avoid limitations such as the binding‐site barrier and poor tissue penetration. While tuning these parameters can improve selectivity and efficacy, immune responses are primarily determined by the physicochemical properties of the carrier and protein corona formation [33]. Overall, a systems‐level approach integrating spatiotemporal control, multivalency and material design is essential to overcome current limitations.

### 3.1 Multivalency for Super‐Selective Targeting

Multivalency refers to the presence of multiple ligands on a nanocarrier surface, either identical or distinct, enabling simultaneous interactions with multiple receptors [34]. This increases local ligand concentration and enhances binding strength and selectivity through combined enthalpic contributions and entropic effects associated with reduced degrees of freedom upon binding [35]. As a result, binding shows a non‐linear dependence on receptor density, with negligible interaction below a threshold and a sharp increase above it. This behaviour allows selective binding based on receptor expression. Super‐selectivity is typically achieved with weak‐affinity ligands, while strong‐affinity ligands reduce selectivity and increase off‐target interactions. Super‐selectivity can be further enhanced through multiplexing, where hetero‐valent ligands are combined to target specific receptor ‘profiles' defined by multiple receptor types and densities. This strategy aligns with phenotypic association theory (PAT), which provides a framework for designing nanocarriers that selectively interact with defined cellular phenotypes [36, 37, 38].

### 3.2 Topology

The avidity of ligand–receptor interactions is governed by the interplay between valency, individual binding affinity and topology. Flexible ligands and receptors increase the number of possible binding configurations, enhancing interaction probability, whereas rigid and short linkers restrict these configurations and reduce binding efficiency. Avidity entropy, reflecting the distribution of ligand–receptor pairings, is a non‐linear function influenced by the number of possible binding configurations and contributes to interaction stability and variability [35]. The role of topology has been demonstrated using DNA‐origami constructs with identical ligand composition but different spatial arrangements, showing that geometric patterning alone can modulate receptor interactions [39].

### 3.3 The Dynamics of Active Targeting

Active targeting is governed not only by binding affinity but also by dynamic processes regulating nanoparticle–cell interactions. At the molecular level, interactions with biomolecules in biological fluids are driven by non‐covalent forces, including electrostatic, hydrophobic, hydrogen bonding and π–π interactions [40]. These interactions are defined by association and dissociation rates, determining binding stability and lifetime. A key dynamic process is the formation of the protein corona, a layer of biomolecules that continuously exchanges depending on the biological environment, altering nanoparticle identity and influencing targeting, biodistribution and uptake. Opsonisation represents a specific outcome of this process, promoting immune recognition and clearance and creating a competition between circulation, targeting and elimination [19]. To mitigate these effects, protein repellent polymers such as PEG are commonly used to introduce steric barriers that reduce non‐specific interactions and regulate ligand accessibility [41]. Receptor expression and availability are also dynamic, governed by internalisation, recycling and lateral diffusion. Biological barrier crossing, including extravasation, blood‐brain barrier transport and renal filtration, involves kinetically controlled processes such as endocytosis, intracellular trafficking and transcytosis. Binding strength plays a critical role: high avidity can enhance binding but may promote lysosomal degradation, whereas moderate avidity seem to favour receptor clustering and transcytosis, enabling more efficient transport [42]. Additionally, the glycocalyx introduces steric effects that modulate receptor accessibility and interaction strength. In our body, the dynamic multi‐step process of targeting is particulary evident in T cell migration across blood vessels [43], which involves rolling, adhesion, crawling and transmigration, all regulated by finely tuned binding kinetics. At the systemic level, nanocarrier transport is governed by hemodynamics and the balance between convection and diffusion [44]. Red blood cell dynamics promote margination towards vessel walls, enhancing endothelial interactions, while particle size influences transport behaviour, with larger particles being convection‐driven and smaller ones diffusion‐dominated. Moreover, crossing into tissues depends on vessel permeability (e.g., fenestrations, inflammation) and interaction dynamics with the endothelium. Nanoparticles may also aggregate or disassemble depending on local conditions (pH, ionic strength, protein adsorption), affecting size and transport behaviour. Therefore, targeting efficiency reflects a balance between transport and binding kinetics. Nanoparticles must reach the target site within relevant physiological timescales, competing with blood flow, immune clearance and non‐specific uptake.

## 4 Future Directions

At present, current active targeting strategies, largely based on ligand–receptor interactions, do not fully capture the complexity of biological systems. Effective targeting requires an integrated design approach that considers not only ligand type and density, but also their spatial organisation, dynamic presentation and the physicochemical properties of the nanocarrier, such as size and surface characteristics. Nanoparticle design must account for the dynamic nature of biological environments and the specific requirements of different biological barriers. In this context, adaptive or stimuli‐responsive systems capable of spatiotemporal control represent a promising direction to enhance targeting precision and reduce off‐target effects. Advances in sequence‐defined polymers and synthetic macromolecules provide powerful tools to address these challenges by enabling precise control over multivalency, steric interactions and dynamic competition at the nano‐bio interface [45]. Modern synthetic strategies, including controlled polymerisation and click chemistry, enable precise control over macromolecular architecture, facilitating the design of systems with tailored properties and improved selectivity [46, 47, 48, 49, 50, 51, 52]. In addition, polymer self‐assembly can be tailored to achieve the selected physicochemical properties of nanocarriers and to dynamically evolve in response to biological interactions at different stages of targeting. Other nanocarriers, besides polymer‐based materials, offer complementary features that can further support dynamic and programmable targeting. Lipid‐based systems, including liposomes and lipid nanoparticles, provide high biocompatibility and membrane fluidity, enabling ligand mobility, fusion processes and stimuli‐responsive behaviour. Hybrid nanocarriers, including biomimetic and cell‐derived components such as EVs or membrane‐coated nanoparticles, introduce intrinsic biological recognition capabilities and improved immune evasion. These platforms can integrate multiple functionalities within a single system, supporting adaptive interactions with biological environments and facilitating efficient navigation across complex barriers. Optimising multivalency, ligand presentation, self‐assembly and particle size may improve targeting while minimising off‐target effects.

Despite these progresses, our ability to predict and control interactions in our body remains limited. Current approaches largely depend on empirical, trial‐and‐error strategies that are resource‐intensive and poorly suited for clinical translation. Typically, they begin with qualitative chemical intuition, prior experience and insights from related nanomaterials, followed by iterative in vitro and in vivo testing. This workflow is costly, constrained by ethical considerations and characterised by high failure rates, ultimately limiting translatability. It also makes the systematic screening of large nanocarrier libraries impractical. Altogether, these challenges have constituted a major bottleneck in the development of nanomedicine and polymer therapeutics over the past two decades.

Future developments may address these limitations by moving beyond empirical approaches and integrating molecular dynamics simulations (MD) with artificial intelligence (AI) to predict interactions more accurately. In this context, a design‐by‐architecture framework, in which targeting rules are encoded directly into (macro)molecular structures, represents a promising strategy for next‐generation nanomedicine. By treating polymer topology and supramolecular organisation as primary design parameters alongside chemical composition, the structural architecture of nanocarriers can be rationally engineered to control their biological behaviour, including ligand presentation and interactions with the protein corona [52]. Such an approach may enable more effective and clinically translatable targeting strategies than those relying solely on conventional surface functionalization. MD simulations enable the investigation of ligand–receptor interactions, conformational flexibility, and nanoparticle self‐assembly at atomistic and mesoscopic scales, providing insight into binding mechanisms and free energy landscapes [35]. Coarse‐grained approaches extend these analyses to larger systems and longer timescales, allowing the study of multivalent interactions, membrane engagement and nanoparticle behaviour in complex environments [53]. In parallel, machine learning approaches are increasingly used to predict structure–property relationships and guide the design of polymer architectures with desired biological functions [54, 55, 56]. When combined with experimental data, these tools may enable the prediction of key processes such as protein corona formation, biodistribution and cellular uptake, supporting a transition towards rational, data‐driven design.

However, the application of these approaches to nanomaterial‐cell targeting and receptor‐level dynamics remains at an early stage [57, 58, 59], and significant challenges persist in accurately capturing the complexity of biological environments. Increasing attention is also being directed towards transport‐related barriers, such as the blood‐brain barrier and dense tumour microenvironments, highlighting the importance of particle navigation, tissue penetration and intracellular trafficking alongside binding.

Looking forward, the integration of polymer chemistry, nanotechnology, computational modelling and cell biology into a unified design‐by‐architecture framework will be essential to uncover the molecular principles governing targeting selectivity. Advances in programmable polymers and nanomaterials may enable the encoding of specific ‘biological instructions' within macromolecular structures, analogous to natural biomolecules. In parallel, bioinspired and hybrid systems, such as EV‐like platforms, offer opportunities to combine natural targeting mechanisms with synthetic tunability. Ultimately, the development of systems capable of dynamic, reversible interactions and temporally controlled signal presentation may enable adaptive targeting strategies that more closely mimic physiological processes. Together, these advances point to a new way of thinking about active targeting, which should be understood not as a static binding event, but as a dynamic, multi‐scale process shaped by molecular recognition, transport and biological context.

## Conflicts of Interest

The author declares no conflicts of interest.

## Data Availability

Data sharing is not applicable to this article as no new data were created or analysed in this study.
